# Atomically Dispersed Fe-Co Bimetallic Catalysts for the Promoted Electroreduction of Carbon Dioxide

**DOI:** 10.1007/s40820-021-00746-9

**Published:** 2021-12-10

**Authors:** Zhangsen Chen, Gaixia Zhang, Yuren Wen, Ning Chen, Weifeng Chen, Tom Regier, James Dynes, Yi Zheng, Shuhui Sun

**Affiliations:** 1 Institut National de la Recherche Scientifique-Énergie Matériaux et Télécommunications, Varennes, QC J3X 1P7 Canada; 2 School of Materials Science and Engineering, University of Science and Technology, 100083 Beijing, People’s Republic of China; 3 Canadian Light Source, University of Saskatchewan, Saskatoon, SK S7N 2V3 Canada; 4 Research Institute of Photocatalysis, State Key Laboratory of Photocatalysis on Energy and Environment, Fuzhou University, Fuzhou, 350116 People’s Republic of China

**Keywords:** CO_2_ reduction, Electrocatalysis, Syngas production, Cobalt, Iron, Bimetallic catalysts

## Abstract

The electroreduction reaction of CO_2_ (ECO_2_RR) requires high-performance catalysts to convert CO_2_ into useful chemicals. Transition metal-based atomically dispersed catalysts are promising for the high selectivity and activity in ECO_2_RR. This work presents a series of atomically dispersed Co, Fe bimetallic catalysts by carbonizing the Fe-introduced Co-zeolitic-imidazolate-framework (C–Fe–Co–ZIF) for the syngas generation from ECO_2_RR. The synergistic effect of the bimetallic catalyst promotes CO production. Compared to the pure C–Co–ZIF, C–Fe–Co–ZIF facilitates CO production with a CO Faradaic efficiency (FE) boost of 10%, with optimal FE_CO_ of 51.9%, FE_H2_ of 42.4% at − 0.55 V, and CO current density of 8.0 mA cm^−2^ at − 0.7 V versus reversible hydrogen electrode (RHE). The H_2_/CO ratio is tunable from 0.8 to 4.2 in a wide potential window of − 0.35 to − 0.8 V versus RHE. The total FE_CO+H2_ maintains as high as 93% over 10 h. The proper adding amount of Fe could increase the number of active sites and create mild distortions for the nanoscopic environments of Co and Fe, which is essential for the enhancement of the CO production in ECO_2_RR. The positive impacts of Cu–Co and Ni–Co bimetallic catalysts demonstrate the versatility and potential application of the bimetallic strategy for ECO_2_RR.

**Supplementary Information:**

The online version contains supplementary material available at 10.1007/s40820-021-00746-9.

## Highlights

- X-ray photoelectron spectroscopy results confirmed the increased number of M–N sites in the bimetallic Fe–Co catalyst.
- Synchrotron-based X-ray absorption fine structure demonstrated that the interaction in the coordination environments of the different transition metal sites facilitated the CO production in electroreduction reaction of CO_2_ (ECO_2_RR).
- This bimetallic strategy has also been extended to fabricate other catalysts such as Cu–Co and Ni–Co, which also exhibited enhanced performance for ECO_2_RR.

**Supplementary Information:**

The online version contains supplementary material available at 10.1007/s40820-021-00746-9.

## Graphical abstract

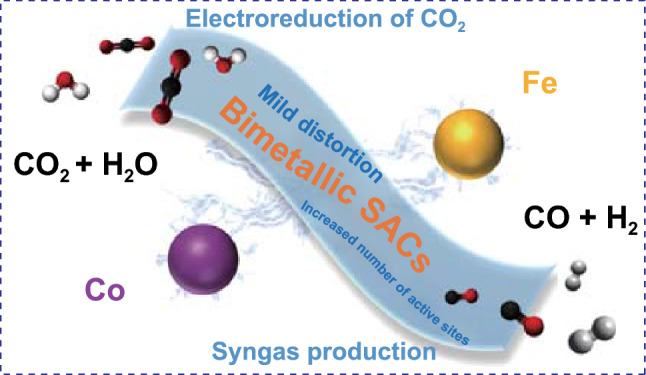

## Introduction

More and more anthropogenic activities depend on the consumption of fossil fuels, which increasingly causes the energy crisis and excessive carbon dioxide (CO_2_) emissions. The latter greatly contributes to the acceleration of global warming. The reduction reaction of CO_2_ (CO_2_RR), i.e., the conversion of CO_2_ into value-added chemicals and fuels, provides a promising alternative to the solution for the energy crisis and global warming [1]. The electroreduction reaction of CO_2_ (ECO_2_RR) contains advantages because of the great potential in future commercialization including renewable energy-generated electricity and easy scale-up. One of the most economic reduction products from ECO_2_RR, carbon monoxide (CO) that is the main component of syngas (gas mixture of CO and H_2_) attracts considerable attention [2]. CO or syngas produced from ECO_2_RR can be applied as feedstock in established industrial processes (e.g., Fischer − Tropsch synthesis) for the production of various important chemicals, such as long-chain hydrocarbons, methanol, higher alcohols, and fuels [3–5]. CO production from ECO_2_RR could contribute to the conception of completing the carbon loop, which could benefit the sustainable development of human society.

To fulfill the industrialization of ECO_2_RR, it requires noble-metal-free-based catalysts to cut the cost while maintaining high catalytic efficiency. The state-of-art ECO_2_RR catalyst design focuses on metal-based catalysts. For example, Cu-based catalysts are exclusively effective for the production of alcohols/hydrocarbons; Ag, Au, and Zn, or most recently the atomically dispersed M–N-C catalysts are efficient for CO production [6, 7]. Single-atom catalysts (SACs) are known for their efficient active sites, maximized utilization of metal atoms, and low cost. Typical SACs, such as Co–N_x_ [8, 9], atomically Ni or Fe-dispersed nitrogen-doped graphene [10, 11], and Fe–N–C [12], have revealed highly efficient CO production in ECO_2_RR attributed to the unique coordination environment of single-atom sites. The direct ECO_2_RR production of syngas with different H_2_/CO ratios could be highly desirable for various downstream products in Fischer − Tropsch synthesis and other thermochemical processes, compared to the traditional industrial methods (e.g., reforming of natural gas and coal, the water gas shift reaction), which demand relatively harsh reaction conditions at high-cost [13, 14]. SACs, such as atomically dispersed Ni catalysts [15], and nickel@nitrogen-doped carbon nanotubes [16], also demonstrate excellent syngas production performance. In recent years, bimetallic catalyst materials in many mainstream reactions have been developed. The hybrid alloy structures of NiCo and FeNi benefit oxygen reduction reaction (ORR) and oxygen evolution reaction (OER), while the synergistic effects between Pd and CoO_x_ promoting CO oxidation [17–19]. The interaction of different metal-sites with the modification of the foreign metal could improve the intrinsic catalytic activity of ECO_2_RR in bimetallic catalysts [20]. Pd/NbN promotes the formation of PdH during the ECO_2_RR process, leading to the enhancement of syngas production [21]. The combinations of TMs Co, Ni, and Fe with noble-metal Ag create the synergism that increases the tenability of syngas ratio in syngas production from ECO_2_RR [22].

The strategy of utilizing noble-metal-free catalyst materials has advantages of the synergistic effects of different metal sites in bimetallic TM-catalysts, the augmented production rate of syngas, and tunable ratios of CO/H_2_. Chen et al. fabricated bimetallic Co, Ni SACs for syngas production from ECO_2_RR [23]. Benefiting from the individual selectivity toward H_2_ and CO for Co and Ni, respectively, the final CoNi bimetallic catalyst exhibited the tunable H_2_/CO ratios of 0.8–1.3 in the reduction product [23]. It is worthwhile for the extensive exploration, especially in the earth-abundant TM elements such as Fe, Co, Ni, and Cu for syngas production with tunable H_2_/CO ratios in ECO_2_RR. The classic bimetallic catalysts are alloy catalysts where two different metals are well intermixed and in close proximity/coordination to each other, thus promoting catalytic performance. The situation is very different in atomically dispersed M–N-C catalysts with different types of metals present. Further researches are ongoing to investigate whether the different M–N-C sites in the bimetallic atomically dispersed catalysts working separately or there is an interaction between them like the alloy catalysts [24]. The objectives of the present study are to design bimetallic atomically dispersed catalysts, investigate the interaction between different metal sites and the contribution to the performance of ECO_2_RR.

Here, we report a bimetallic catalyst with atomically dispersed Co and Fe sites for the highly efficient ECO_2_RR for syngas production, which is our preliminary attempt to understand the interaction between Co and Fe and its contribution to the catalytic performance. The bimetallic catalysts are fabricated by using a zeolitic imidazolate framework (ZIF-8) as the precursor, which is a metal–organic framework (MOF) that is tunable in metal nodes [8]. The Co-ZIF is first constructed on ZIF precursor, followed by the introduction of Fe. The introduction of Fe into Co–ZIF (Fe–Co–ZIF) creates the interaction between Co and Fe sites in the final carbonized catalysts (C–Fe–Co–ZIF-*x*, *x* is the adding amount of Fe (wt%) in the ZIF precursor). X-ray photoelectron spectroscopy (XPS) illustrates that the introduction of Fe with the proper amount could increase the number of M–N sites in the catalyst, promoting the ECO_2_RR performance. The interactions of different TMs in the bimetallic catalysts are indicated by X-ray absorption spectroscopy (XAS). It confirms that the coordination environment of Co is distorted by the addition of Fe in the bimetallic catalysts. Electrochemical measurements are carried out to investigate the catalytic performance of C–Fe–Co–ZIFs. C–Fe–Co–ZIF-1.6 wt%-Fe that has the mild distortions in Co–N and Fe–N sites exhibits the best ECO_2_RR performance toward CO production while maintaining the high total FE_CO+H2_ of around 93% for more than 10 h. Cu–Co and Ni–Co bimetallic catalysts (carbonized Cu and Ni-modified Co–ZIF) are confirmed to be able to promote ECO_2_RR performance, demonstrating the versatility of the bimetallic SACs strategy. This work systematically investigates the interaction between different metals in bimetallic atomically dispersed catalysts for ECO_2_RR, providing interesting insights into the design of the catalyst for the next generation.

## Experimental Section

### Instruments

The X-ray diffraction (XRD) patterns were collected on an X-ray diffractometer (Bruker D8 Advance) with a CuKα X-ray source (*λ* = 1.542 Å) and a scintillator detector. Scanning electron microscopy (SEM) images were obtained using a focused ion beam and scanning electron microscope (Tescan LYRA 3 XMH) at 20 kV. Transmission electron microscopy (TEM) was performed on a JEOL ARM200F operated at 200 kV. For atomic resolution imaging, the measurements were performed under HAADF-STEM mode. Samples for TEM were prepared by drop-drying the samples from their diluted ethanol suspensions onto carbon-coated copper grids. Inductively coupled plasma-optical emission spectrometry (ICP-OES) results were obtained by an Agilent 5100 ICP-OES. XPS experiments were undertaken on a VG Escalab 220i XL using monochromatic 1486.6 eV Al Kα radiation. The peak energies were calibrated by placing the graphite C 1 s peak at 284.8 eV. The spectra were fitted with mixed Gaussian–Lorentzian component profiles after a Shirley background subtraction by CasaXPS software. X-ray absorption spectroscopy including X-ray absorption near-edge spectra (XANES) and extended X-ray absorption fine structure (EXAFS) at Co K-edge and Fe-K-edge were collected in total-fluorescence-yield mode using a 32-element Ge detector at ambient condition on the 06ID-1 Hard X-ray MicroAnalysis (HXMA) beamline at the Canadian Light Source (CLS). During the data collection, the CLS storage ring (2.9 GeV) was operated under 250 mA mode and the HXMA superconducting wiggler was run at 1.9 T. The scan range was kept in an energy range of 7510–8350 eV for Co K-edge and 6910–7755 eV for Fe K-edge. Data collection configuration was using metal Co and Fe foils as energy calibrations by in step calibration for every data set. The baseline of pre-edge was subtracted and the post-edge was normalized in the spectra. EXAFS analysis was conducted using Fourier transform on k^3^-weighted EXAFS oscillations to evaluate the contribution of each bond pair to Fourier transform peak. The XANES of N K-edge, Co L-edge, and Fe L-edge were measured in total X-ray electron yield mode at room temperature on the 11ID-1 High-Resolution Spherical Grating Monochromator (SGM) beamline at the CLS, while the samples were subjected in an ultrahigh vacuum chamber. Pure metals such as Co and Fe were used as the reference to calibrate the energy for every data set.

### Electrochemical Measurements

Catalyst electrodes were prepared by dropping the catalyst ink onto the carbon paper (Sigracet 25 BC) with a fixed area of 1 cm^2^. The catalyst ink was prepared by mixing 0.5 mg of the catalyst powder, 120 μL of DI water, 120 μL of ethanol, and 2 μL of Nafion® perfluorinated resin solution (5 wt%, Sigma). The mixture was treated with ultrasound for 30 min and dropped onto the carbon paper on an 80 ℃ hot plate. The electrode was finally dried under 70 ℃ in an oven for further experiments.

Cyclic voltammetry (CV, performed at the scan rate of 20 mV s^−1^), linear sweeping voltammetry (LSV, performed at the scan rate of 5 mV s^−1^), the chronoamperometry test, double-layer capacitance, and the electrochemical impedance spectroscopy (EIS, performed at open circuit potential with a high frequency of 100,000 and low frequency of 0.01) were carried out in a custom-made two-chamber H-type cell on a CHI 760D electrochemical workstation with the catalyst electrode as the working electrode. Working and reference electrodes were fixed in one chamber and the counter electrode was fixed in the other chamber. A proton exchange membrane (Nafion™ N115) separated the two chambers of the H-cell. The reference electrode was an Ag/AgCl electrode with a saturated KCl filling solution. The counter electrode was a Pt wire. Potential versus reversible hydrogen electrode (RHE) was calculated as E versus RHE = E versus Ag/AgCl + 0.197 V + 0.0592 V × pH. The pH values of CO_2_ and N_2_-saturated 0.5 M KHCO_3_ electrolyte used in this work are 7.23 and 8.36, respectively. Unless notified elsewhere, the automatic iR compensation (80%) was applied to all the measurements.

During the electrochemical tests, each chamber of the H-cell was filled with 40 mL 0.5 M KHCO_3_ electrolyte. CO_2_ (Praxair, 99.99%) (or N_2_ (Praxair, 99.999%) for the blank experiments) was bubbled through the electrolyte in the working electrode chamber with a flow rate of 20 standard cubic centimeters per minute (SCCM) at least 30 min before the tests. The gas outlet was introduced into a gas-sampling loop of the gas chromatography (GC; 9790II, Fuli) for the quantification of CO and H_2_. The GC was equipped with a packed HaySep A column, a packed MolSieve 5 A column, and a packed Porapak N column with argon (Praxair, 99.999%) as the carrier gas. A thermal conductivity detector (TCD) was used to qualify and quantify H_2_. A flame ionization detector coupled with a Ni reformer was used to qualify and quantify CO. For the Faradaic efficiency (FE) tests, the product gas was tested in GC after 15 min of the reaction at each set potential.

The product gas was introduced into GC to test the FE of CO and H_2_.

The calculation of FE follows Eq. 1:

1 $$\mathrm{FE}=\frac{nF{C}_{i}vP}{jRT}$$

where *n* is the number of electrons involved; *F* as the Faradaic constant (96,485.33289 C mol^−1^); ${C}_{i}$ as the volume fraction of a certain product determined by online GC; $v$ = 20 sccm (3.3 × 10^–7^ m^3^ s^−1^); *P* = 101,300 Pa; *j* = Total current (A); *R* as the gas constant of 8.314 J mol^−1^ K^−1^; *T* = 293 K.

The current densities of CO (${j}_{\mathrm{co}}$) and H_2_ (${j}_{H2}$) are calculated based on Eq. 2:

2 $${j}_{\mathrm{co}(H2)}={j}_{\mathrm{total}}\times {\mathrm{FE}}_{\mathrm{co}(H2)}$$

The total turnover frequency (TOF) for CO and H_2_ was calculated by assuming all the active sites were M-N_4_ sites and based on Eq. 3:

3 $$\mathrm{TOF}=\frac{{j}_{\mathrm{co}(H2)}\times {A}_{\mathrm{electrode}}/{2\times 1.6*10}^{-19}}{{M}_{\mathrm{sample}}{N}_{\%}{MN}_{\%}{N}_{A}/4\times {M}_{N}}$$

where ${j}_{\rm co(H2)}$ is the current density of CO (${j}_{\mathrm{co}}$) or H_2_ (${j}_{\rm H2}$), ${A}_{\rm electrode}$= 1 cm^−2^ is the electrode geometric area, ${M}_{\rm sample}$= ${5\times 10}^{-4}g$ is the mass of catalyst on the electrode, ${N}_{\%}$ is the mass ratio of Nitrogen in the catalyst obtained from XPS, ${MN}_{\%}$ is the metal-N content ratio obtained from the XPS deconvolution results of N 1 s, ${N}_{A}$ = ${6.02\times 10}^{23}$ is Avogadro constant, ${M}_{N}$ is the atomic mass of nitrogen.

The Tafel slope was calculated based on the Tafel equation (Eq. 4):

4 $$\eta =a+b\,\mathrm{log}{j}_{CO}$$

where *ƞ* is the overpotential, *b* is the Tafel slope, and ${j}_{CO}$ is the current density for CO formation.

## Results and Discussion

### Preparation and Characterization of Catalysts

**Fig. 1:**
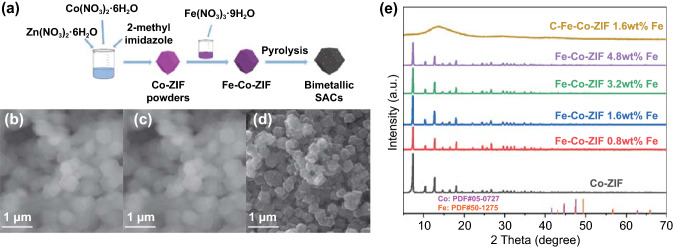
Structural characterization of C–Fe–Co–ZIF catalysts. **a** A fabrication schematic of the C–Fe–Co–ZIF catalysts; SEM images of **b** Co-ZIF. **c** Fe-Co-ZIF-1.6 wt%-Fe. **d** C–Fe–Co–ZIF-1.6 wt%-Fe at scale bar of 1 μm. **e** XRD patterns of Fe-Co-ZIF series catalysts with different Fe adding amounts and the carbonized Fe-Co-ZIF-1.6 wt%-Fe (C–Fe–Co–ZIF)

The one-pot synthesis strategy of mixing all the ingredients led to a relatively small product yield (which could not be collected). The two-step synthesis method is employed to fabricate the atomically dispersed bimetallic catalysts. The synthetic procedure for the bimetallic Co–Fe catalysts is illustrated in Fig. 1a. The Fe–Co–ZIF precursors are prepared with the impregnation method to modify ZIF-8 into Co–ZIF [8] and absorb Fe source (detailed in supporting information). Through this two-step synthesis method, the introduction of Fe does not interfere with the crystallization of Co–ZIF particles, resulting in the success of the yield of the bimetallic Fe–Co–ZIF precursors. Combined with the pyrolysis process to carbonize the ZIF particles and vaporization of Zn, atomically dispersed Co, Fe bimetallic catalysts are finally fabricated. SEM images reveal that the series of Fe–Co–ZIFs retain the morphology of the original Co–ZIFs with the various amount of Fe added in the synthesis (Figs. 1b, c, and S1). After the pyrolysis process, the carbonized Fe–Co–ZIF (C–Fe–Co–ZIF) catalysts retain the morphology of Fe–Co–ZIFs (Fig. 1c, d). C–Fe–Co–ZIF also has better conductivity than ZIF precursors, providing improved image quality in Fig. 1d than Fig. 1b, c. XRD measurements show that Fe–Co–ZIFs keep the same crystalline structure as that of Co-ZIF, without any characteristic peak assigned to Co and Fe crystals (Fig. 1e). The absence of Co and Fe in XRD patterns can be due to the extremely low content of the metal element in the final catalysts (ICP indicates: 2.2 wt% of Co and 1.0 wt% of Fe in C–Fe–Co–ZIF-4.8 wt%-Fe). The XRD pattern of C–Fe–Co–ZIF (Fig. 1e) demonstrates that the final bimetallic catalysts appear as amorphous carbon material.

**Fig. 2:**
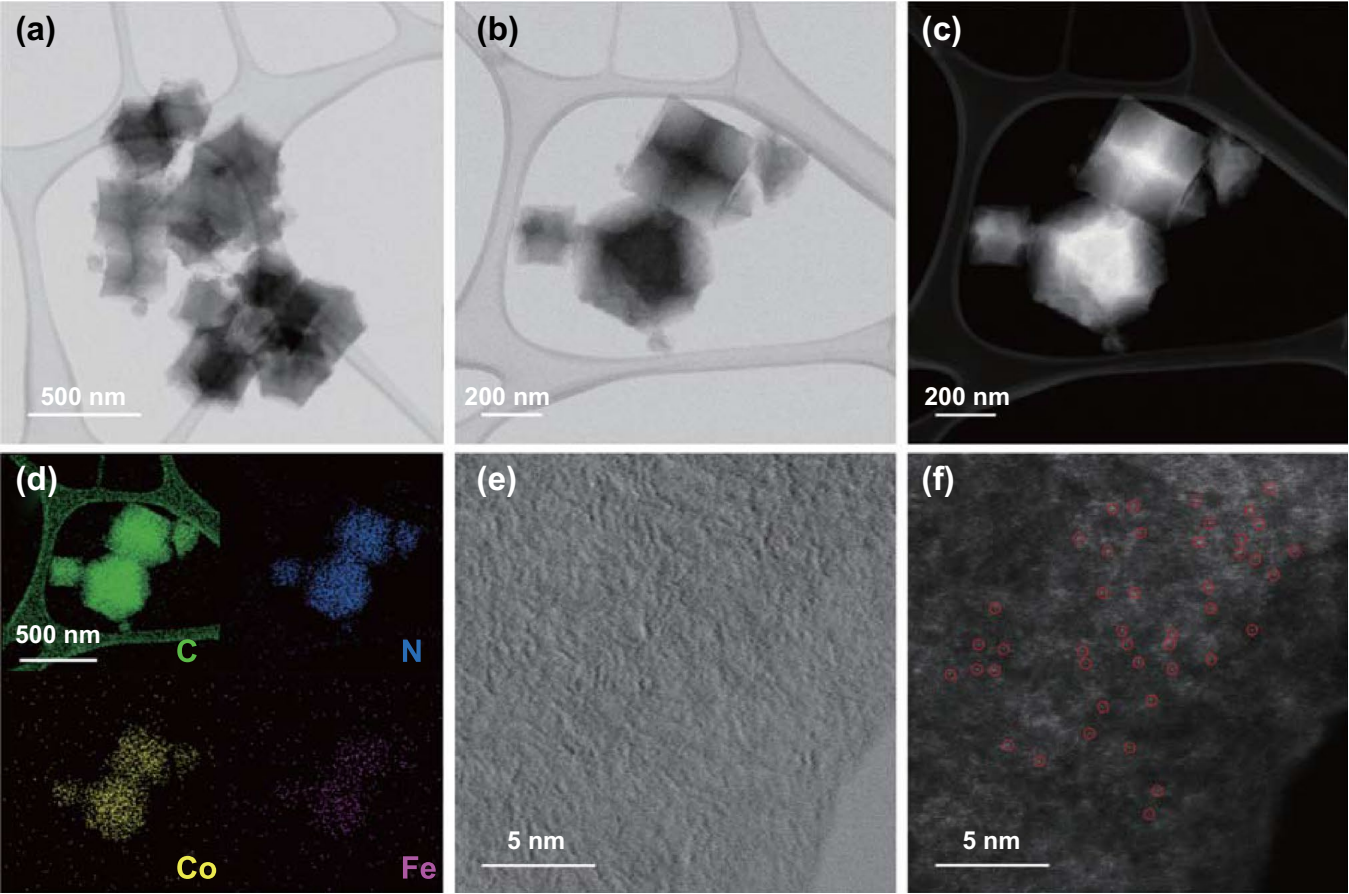
**a, b** TEM images, **c** HAADF-STEM images, **d** elemental mapping images, **e** magnified TEM images, **f** atomic-resolution HAADF-STEM images of C–Fe–Co–ZIF-1.6 wt%-Fe powders

TEM images show that the C–Fe–Co–ZIF particles are around 300 nm (Fig. 2a–c), which agrees with the SEM results. The energy-dispersive X-ray (EDX) spectroscopy elemental mappings depict the distributions of Co, Fe, C, and N, indicating that Co and Fe atoms are evenly distributed in C–Fe–Co–ZIF (Fig. 2d). Aberration-corrected high-angle annular dark-field scanning transmission electron microscopy (HAADF-STEM) with the sub-angstrom resolution are employed to directly observe the atomic dispersion of the metal atoms, benefiting from higher Z-contrast of Co and Fe than N and C (Fig. 2e, f). In Fig. 2f, the single atom of Co and Fe (tiny bright spots) are well dispersed in C–Fe–Co–ZIF 1.6 wt% Fe. To further demonstrate so, we have also conducted the X-ray absorption spectroscopy analysis, and indeed, we found the existence of the peaks for single-atom bindings in EXAFS (please see the corresponding discussion later on in this work). In short, the HAADF-TEM and EXAFS results are auxiliary to support the conception of single atoms in the catalysts. Other than atomically dispersed metal atoms, small metal clusters are also found in the bimetallic catalysts (Fig. S2). It illustrates the co-existence of the single-metal-atoms and metal clusters in the C–Fe–Co–ZIF.

**Fig. 3:**
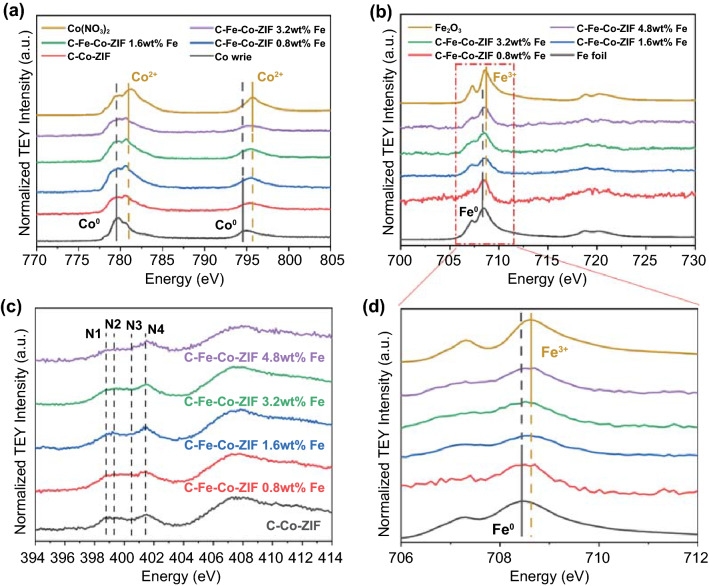
**a** Co L-edge XAS. **b** Fe L-edge XAS. **c** N K-edge XAS. **d** Enlarged Fe L-edge XAS of C–Fe–Co–ZIF samples

Soft X-ray (metal L-edge) is attributed to the excitement of the 3d valence states. And the total electron yield mode (TEY) of metal L-edge is a surface-sensitive technique, which could be a more sensitive and direct probe for the metal oxidation states on the surface of the catalyst. The Co L-edge X-ray absorption spectroscopy (XAS) is shown in Fig. 3a. In the region at ca. 795 V, the peaks for C–Fe–Co–ZIF are located between Co wire and Co(NO_3_)_2_. It suggests that the valences of Co atoms in C–Fe–Co–ZIF catalysts are between 0 to + 2. There are two main peaks in the region from 778 to 782 V. Co wire (Co^0^) is dominated by the peak located before 780 V, while Co(NO_3_)_2_ (Co^2+^) is dominated by the peak located after 780 V. All C–Fe–Co–ZIFs have the relatively close intensity for these two peaks while the intensity of the peak after 780 V is slightly higher than that of the peak before 780 V, indicating the valences for the C–Fe–Co–ZIF samples are close to + 2. Fe L-edge XAS in Fig. 3b demonstrates that all the C–Fe–Co–ZIFs samples share the same peak pattern. The peak at ca. 707 and 709 eV can be ascribed to *t*_2g_ and *e*_g_ states, respectively [25]. The enlarged region in Fig. 3d indicates that the valences of Fe atoms in C–Fe–Co–ZIF catalysts are between 0 to + 3. The results agree well with previous literature that TMs in SACs are usually in an oxidation state [26, 27]. Furthermore, N sites in the catalysts are investigated from N K-edge XAS (Fig. 3c). Two absorption edges at around 400 eV and 407 eV are corresponding to 1 *s* → π* and 1 *s *→ σ* transitions, respectively [28]. The wide peaks at ca. 407 eV show no obvious changes in all samples. In the 1 *s* → π* edge, the peaks at ca. 401.5 eV (N4) appear for all the samples, which can be assigned to graphitic N. Almost all the samples reveal a plateau in the range from 398.5 to 400.5 eV, where peaks at ca. 398.8 (N1), 399.2 (N2), and 400.5 (N3) eV can be assigned to pyridinic N, metal-N bindings, and pyrrolic N, respectively [12, 29]. The graphitic N, pyridinic N, and pyrrolic N sometimes could facilitate the ECO_2_RR process but are regarded as less effective than the metal-centered sites [11, 30]. The contents of metal-N binding directly reflect the property of M-Nx active sites in SACs, which are vital to catalytic reactions such as ECO_2_RR [31–33]. C–Fe–Co–ZIF-1.6 wt%-Fe possesses the sharpest peak in the N2 region, which represents the optimal metal-N bindings. It implies the higher metal-N content of C–Fe–Co–ZIF-1.6 wt%-Fe than other samples.

**Fig. 4:**
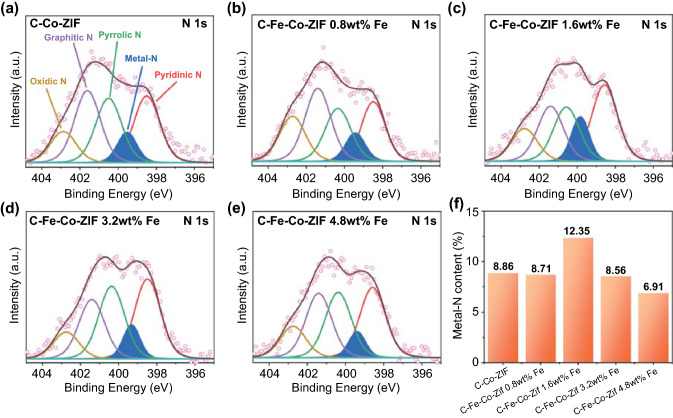
N 1 s XPS of **a** C–Co-ZIF, **b** C–Fe–Co–ZIF-0.8 wt%-Fe, **c** C–Fe–Co–ZIF-1.6 wt%-Fe, **d** C–Fe–Co–ZIF-3.2 wt%-Fe, **e** C–Fe–Co–ZIF-4.8 wt%-Fe. **f** The percentage of metal-N content among N species of C–Fe–Co–ZIF samples

XPS could characterize the specific species on the surface of the material. Although it is challenging to accurately identify the species solely by a single XPS, the combination of the two auxiliary techniques (e.g., XPS and XAS) can strongly support one opinion. Thus, we further verified the N K-edge results from XAS, by XPS. The XPS of N 1 s in Fig. 4a-e confirms the ascription of pyridinic N, metal-N, pyrrolic N, and graphitic N. Notably, The percentage comparison of metal-N content among all the N species for C–Fe–Co–ZIF in Fig. 4f shows that the metal-N content increases significantly for C–Fe–Co–ZIF-1.6 wt%-Fe, which is consistent with the XAS results. It suggests that C–Fe–Co–ZIF-1.6 wt%-Fe could contain the maximum active sites for ECO_2_RR. As the Fe content continuously increases in the catalyst precursor, however, the metal-N content decreases in the C–Fe–Co–ZIF. It is possibly due to the generation of the metal clusters when too much Fe is added, that the aggregation hinders the formation of metal-N bindings.

**Fig. 5:**
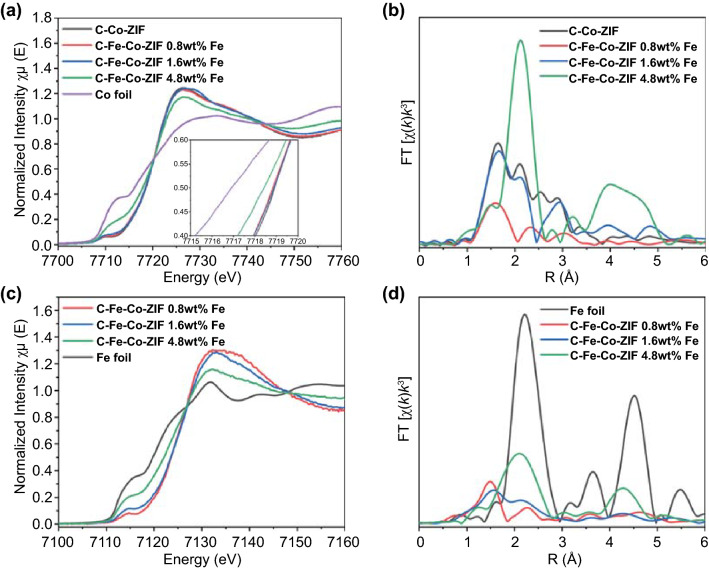
**a** Co K-edge XANES, inset: enlarged region of the white line. **b** Co EXAFS. **c** Fe K-edge XANES. **d** Fe EXAFS of C–Fe–Co–ZIF samples

Hard X-ray (metal K-edge) that is bulk sensitive could not only provide information (XANES) on the metal oxidation states but also give insights into the local coordination environments (from EXAFS), which is essential for the SAC characterization. XANES of Co in Fig. 5a further reveals that gradually increasing the introduction of Fe reduces the valence of Co in the catalysts (shift to the low energy side). The valences of Co atoms in C–Fe–Co–ZIF-0.8 wt%-Fe and 1.6 wt%-Fe are the closest to that of C–Co-ZIF. In addition, the pre-edge structure at ca. 7711 eV increases with the increase in Fe in the samples. This pre-edge peak belongs to the quadrupole electron transition of Co 1 s to Co 3d, which reflects the density of empty Co 3d orbitals via hybridization [34]. For C–Fe–Co–ZIF-4.8 wt%-Fe, the pre-edge peak shifts toward that of the Co foil. During the synthesis, only a small amount of Fe is added into Co-ZIF. It is not likely that Co particles are formed because of the increasing metal content of Fe in the final catalyst. Thus, the increased intensity of the pre-edge peak indicates that the adding of Fe affects the electronic structure of Co. The XANES pattern tends to be close to that of Co foil when the Fe content is increased. It implies that Co elements are gradually turning into metallic states with the addition of Fe.

EXAFS is conducted to investigate the coordination environment of Co sites. As shown in Fig. 5b, the C–Co-ZIF sample exhibits a prominent peak at ca. 1.6 Å, which can be ascribed to the Co–N, Co–O, and Co–C coordination, indicating the single-atom state of Co atoms. Several previous studies confirm that metal-N bonds dominate similarly as the peak in ZIF-8 derived SACs [8, 33, 35]. Combined with the XPS results, this peak could be plausibly assigned to Co–N bindings. The peak at ca. 2.1 Å is close to the main peak of Co foil (Fig. S3), which can be ascribed to metal–metal bindings, i.e., the Co–Co coordination. It indicates that C–Co–ZIF consists of atomically dispersed Co atoms as well as Co clusters, which agrees with the TEM results. With a relatively small amount of Fe, the EXAFS *R* space of C–Fe–Co–ZIF-0.8 wt%-Fe shows a dramatic decrease in peak intensities, implying either the significant decrease in coordination number or the intense distortion of the coordination environment of Co atoms. The main peaks of C–Fe–Co–ZIF-0.8 wt%-Fe are located at ca. 1.5 Å and ca. 2.3 Å, which is not identical to that of C–Co–ZIF. It suggests that the Fe atoms greatly affect the coordination environment of Co sites in C–Fe–Co–ZIF-0.8 wt%-Fe. With the increase in Fe content, the *R* space peaks of C–Fe–Co–ZIF-1.6 wt%-Fe tend to be similar to those of C–Co-ZIF with different intensities of the main peaks. It indicates that in the C–Fe–Co–ZIF-1.6 wt%-Fe, the coordination environment of Co sites is less affected by the introduction of Fe than that of C–Fe–Co–ZIF-0.8 wt%-Fe. With large content of Fe, C–Fe–Co–ZIF-4.8 wt%-Fe exhibits a prominent peak at ca. 2.1 Å that belongs to metal–metal bindings, ascribed to Co–Fe or Fe–Fe coordination. It implies that the large amount of Fe could greatly affect the coordination environment of Co sites (C–Fe–Co–ZIF-4.8 wt%-Fe).

The above methodology is applied for the analysis of Fe atoms. XANES of Fe in Fig. 5c reveals that the gradually increased amount of Fe reduces the valence of Fe in the catalysts, while the valences of Fe in C–Fe–Co–ZIF-0.8 wt%-Fe and C–Co–ZIF-1.6 wt%-Fe are similar. The near edge structure at ca. 7115 eV is assigned to 1 s to 3d transitions [36]. With the increasing adding amount of Fe, the XANES tends to become similar to that of Fe foil, implying the agglomeration of Fe atoms. The coordination environment of Fe sites is investigated by EXAFS (Fig. 5d). The prominent peak at ca. 1.5 Å of C–Fe–Co–ZIF-0.8 wt%-Fe is ascribed to Fe–N bindings (similar interpretation as Co–N), indicating the dominant single-metal-atom state of Fe atoms. With the increasing amount of Fe, the dominant Fe–N peak for C–Fe–Co–ZIF-1.6 wt%-Fe shifts a little to ca. 1.6 Å with a decreased intensity, indicating the distortion to the coordination environment of Fe–N in the catalyst. The peaks at ca. 2.1 Å are observed for C–Fe–Co–ZIF-1.6 wt%-Fe and C–Fe–Co–ZIF-4.8 wt%-Fe. This peak location is not identical to the Fe–Fe binding of Fe foil (2.2 Å) but the same as the one in the previous Co *R* space analysis (Fig. 5b), supporting the assumption of the Co–Fe coordination in the Fe–Co bimetallic catalyst samples.

### Catalytic Performance of the C–Fe–Co–ZIFs for ECO_2_RR

**Fig. 6:**
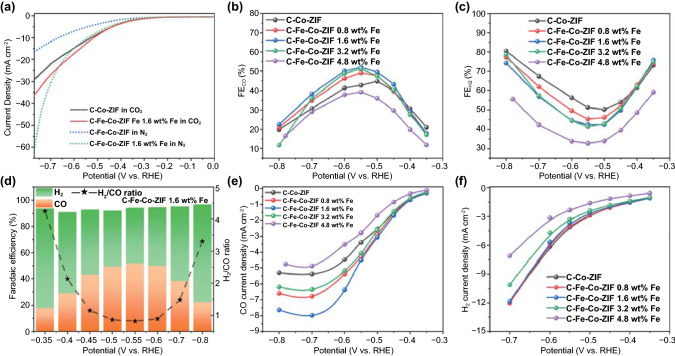
Evaluation of the electrocatalytic performance of the samples. **a** LSV curves of C–Co-ZIF and C–Fe–Co–ZIF-1.6 wt%-Fe in N_2_ or CO_2_-saturated 0.5 M KHCO_3_ solution at a scan rate of 5 mV s^−1^. **b** CO Faradaic efficiency of the catalysts at various applied potentials. **c** H_2_ Faradaic efficiency of the catalysts at various applied potentials. **d** Bars: FE_CO_ and FE_H2_; Stars: H_2_/CO ratio of C–Fe–Co–ZIF-1.6 wt%-Fe at various applied potentials. **e** CO current density of the catalysts. **f** H_2_ current density of the catalysts

The ECO_2_RR evaluations are performed in a custom-made H-cell. Several cycles of CV are pre-run before the electrochemical measurements to stabilize the catalyst electrode. Both CV curves in N_2_ and CO_2_ atmospheres for different C–Fe–Co–ZIF catalysts are measured (Fig. S4). LSV curves of C–Fe–Co–ZIF-1.6 wt%-Fe are better than those of the pure C–Co–ZIF, in both N_2_ and CO_2_ atmospheres (Figs. 6a and S4). Compared to the LSV curve in N_2_, C–Fe–Co–ZIF-1.6 wt%-Fe shows an enhanced current response in the CO_2_ atmosphere in the range of around − 0.35 to − 0.65 V versus RHE. The crossing of CV curves in N_2_ and CO_2_ indicates the production rates of CO and H_2_ are dependent, implying the tunable ratio of CO/H_2_ by adjusting the potential.

FE results in Fig. 6b, c reveal that FE_CO_ and FE_H2_ are in the opposite trend for all the catalysts. C–Fe–Co–ZIF-0.8 wt%-Fe, C–Fe–Co–ZIF-1.6 wt%-Fe, and C–Fe–Co–ZIF-3.2 wt%-Fe exhibit higher FE_CO_ and lower FE_H2_, compared to those of C–Co–ZIF, indicating the promoted CO production in the Co, Fe bimetallic catalyst during the ECO_2_RR process. C–Fe–Co–ZIF-1.6 wt%-Fe and C–Fe–Co–ZIF-3.2 wt%-Fe have similar FE_CO_ and FE_H2_, with FE_CO_ of around 52% (about 10% higher than that of pure C–Co-ZIF) and FE_H2_ around 42% at − 0.55 V versus RHE. The ratio of H_2_/CO can be tuned from 0.8 to 4.2 at different potentials while maintaining the total FE_CO+H2_ above 90% (Fig. 6d). Most of the C–Fe–Co–ZIFs exhibit a higher CO current than that of C–Co–ZIF. C–Fe–Co–ZIF-1.6 wt%-Fe has the highest CO current density of 8.0 mA cm^−2^ at − 0.7 V versus RHE (Fig. 6e). Negligible CO is detected under the controlled electrochemical experiments (in N_2_ atmosphere), confirming the direct CO production from the gaseous CO_2_ during ECO_2_RR. The H_2_ current densities of C–Fe–Co–ZIF-0.8 wt%-Fe and C–Fe–Co–ZIF-1.6 wt%-Fe are very close to that of C–Co-ZIF while C–Fe–Co–ZIF-3.2 wt%-Fe and C–Fe–Co–ZIF-4.8 wt%-Fe exhibit decreased H_2_ current densities (Fig. 6f). Notably, C–Fe–Co–ZIF-4.8 wt%-Fe shows decreases in the production activity in both CO and H_2_ with a total FE_CO+H2_ of around only 70% (Fig. S5), implying the more adding amount of Fe possibly leads to other product formations instead of CO and H_2_.

**Fig. 7:**
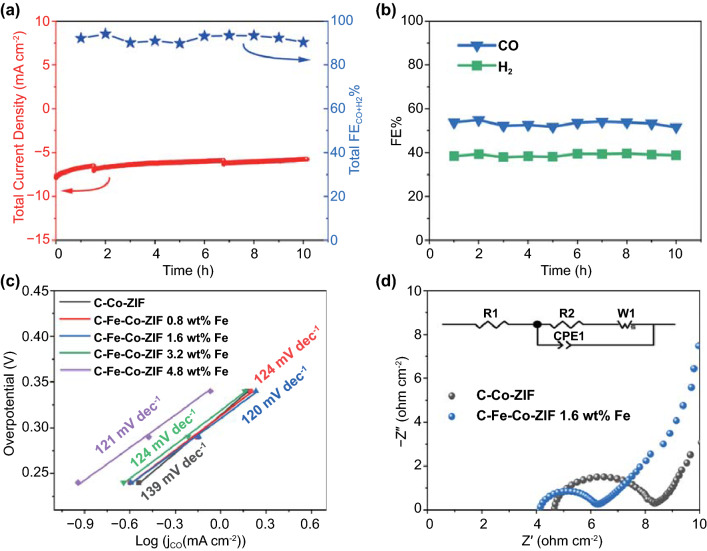
Chronoamperometry test of **a** Total current density and total FE_CO+H2_, **b** FE_CO_ and FE_H2_ of C–Fe–Co–ZIF-1.6 wt%-Fe at -0.55 V versus RHE under CO_2_ atmosphere for the stability evaluation. **c** Tafel slopes of C–Fe–Co–ZIF catalyst electrodes with different Fe adding amounts. **d** EIS results of C–Co-ZIF and C–Fe–Co–ZIF-1.6 wt%-Fe

The chronoamperometry of C–Fe–Co–ZIF-1.6 wt%-Fe in Fig. 7a shows that, during the 10 h of reaction, the total current density drops quickly (10% drop) in the first hour. Then, the decay of the current density is relatively stable, with the maintenance of over 85% of the initial current density for 9 h. The explosion of the gas bubbles generated on the catalyst electrode during the reaction could cause the cliffs in the current curve. FE of CO remains stable for more than 10 h at 52–53% as well as the FE of H_2_ at around 40% (Fig. 7b), which is better than several hours presented in many published reports [23, 37–39].

### Mechanism Investigation

**Fig. 8:**
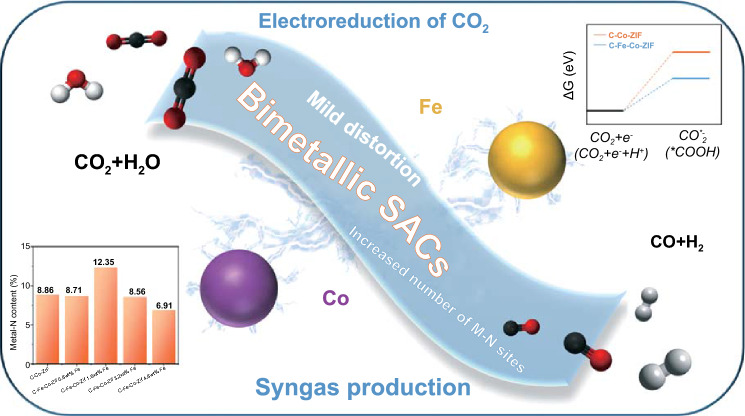
Schematic reaction mechanism of bimetallic catalysts for ECO_2_RR

C–Fe–Co–ZIF-1.6 wt%-Fe has the best CO production activity of ECO_2_RR. We compare the Tafel slope values to gain insights into the CO formation during ECO_2_RR. The schematic of bimetallic Fe-Co catalysts for ECO_2_RR is illustrated in Fig. 8. Tafel slopes of C–Fe–Co–ZIF catalysts are similar in Fig. 7c, of around 120 mV dec^−1^, which is close to the theoretical value of 118 mV dec^−1^ for the first electron-transfer step, suggesting the first electron-transfer step (${\rm CO}_{2}+{\rm e}^{-}\to {\rm CO}_{2}^{*-}$ or ${\rm CO}_{2}+{\rm e}^{-}+{\rm H}^{+}\to {}^{*}{\rm COOH}$) could be the rate-decided step [12]. Compared to the original C–Co-ZIF has a value of 139 mV dec^−1^ (much higher than that of C–Fe–Co–ZIF catalysts, Fig. S6), the improvement of the CO production in ECO_2_RR is caused by the adding of Fe for C–Fe–Co–ZIF catalysts. The more positive initial point of the Tafel slope represents the higher current density. With the more adding amount of Fe in the catalyst (C–Fe–Co–ZIF-4.8 wt%-Fe), the initial point of the Tafel slope shifts significantly toward a negative value, indicating decreased production rate of CO. The double-layer capacitance is employed to estimate the electrochemically active surface area (ECSA) of different catalyst electrodes (Fig. S7). Except for C–Fe–Co–ZIF-4.8 wt%-Fe that has a decreased ECSA, all the other C–Fe–Co–ZIF possess similar ECSA as C–Co-ZIF, implying the enhancement of the CO production for C–Fe–Co–ZIF catalysts comes from the intrinsic properties of the bimetallic catalysts. TOF of the catalysts is calculated based on Eq. 3, as shown in Table 1. At − 0.7 V versus RHE, C–Fe–Co–ZIF catalysts show higher ${\mathrm{TOF}}_{\rm CO}$ values than that of C–Co-ZIF, which agrees with the Tafel slope results. In the meantime, C–Fe–Co–ZIF-0.8 wt%-Fe has the highest ${\mathrm{TOF}}_{\rm CO}$ and ${\mathrm{TOF}}_{\rm H2}$ values among all the catalysts, indicating the strong interaction between Fe and Co atoms (see the previous discussion of XAS) in the catalysts can promote the CO and H_2_ productions during ECO_2_RR.

**Table 1:** TOF of the catalysts at -0.7 V versus RHE

	C–Co-ZIF	C–Fe–Co–ZIF-0.8 wt%-Fe	C–Fe–Co–ZIF-1.6 wt%-Fe	C–Fe–Co–ZIF-3.2 wt%-Fe
${\mathrm{TOF}}_{\rm CO}$(${\rm site}^{-1}{s}^{-1}$)	0.91	1.3	1.2	1.1
${\mathrm{TOF}}_{\rm H2}$(${\rm site}^{-1}{\rm s}^{-1}$)	2.0	2.3	1.8	1.8

Consistently to all characterizations, C–Fe–Co–ZIF-1.6 wt%-Fe exhibits both increased M–N sites and mild distortions in coordination environments of Co–N and Fe–N, leading to the optimum ECO_2_RR performance. The electrochemical impedance spectroscopy (EIS, Figs. 7d and S8) reveals that C–Fe–Co–ZIF-1.6 wt%-Fe exhibits an almost 2 times lower charge-transfer resistance than that of C–Co–ZIF (2.085 and 3.691 Ω for C–Fe–Co–ZIF-1.6 wt%-Fe and C–Co-ZIF, respectively, Fig. S8 and Table S1). The facilitated CO_2_ activation, increased number of M–N sites, and high charge transfer rate attribute to the improved ECO_2_RR performance for Fe-Co-ZIF catalysts (Fig. 8).

### Applications of C–Cu–Co–ZIFs and C–Ni–Co–ZIFs for ECO_2_RR

A series of Cu–Co and Ni–Co bimetallic catalysts based on the same strategy of C–Fe–Co–ZIFs are synthesized to verify the versatility of atomically dispersed bimetallic method of TM elements in ECO_2_RR. The Cu–Co–ZIFs/carbonized Cu–Co–ZIF (C–Cu–Co–ZIF) and Ni–Co–ZIFs/carbonized Ni–Co–ZIF (C–Cu–Co–ZIF) have the same morphology and crystalline structure as that of Fe–Co–ZIF/C–Fe–Co–ZIF (Figs. S9 and S10). The interactions between Cu and Co or Ni and Co are confirmed by XANES and EXAFS (Fig. S11). Similar to C–Fe–Co–ZIF samples, C–Cu–Co–ZIF samples also exhibit a positive enhancement in CO production (Fig. S12). C–Cu–Co–ZIF-3.2 wt%-Cu exhibits the FE_CO_ of around 51% and FE_H2_ of around 37% at − 0.55 V versus RHE. The CO current density reaches 8.3 mA cm-2 at − 0.7 V versus RHE. In contrast, C–Ni–Co–ZIF reveals a negative impact on CO production (Fig. S13). C–Ni–Co–ZIF-3.2 wt%-Ni exhibits the FE_CO_ of around 35% and FE_H2_ of around 29% at − 0.55 V versus RHE. The CO current density reaches 4.7 mA cm^−2^ at − 0.7 V versus RHE. It is noted that the total FE_CO+H2_ of C–Ni–Co–ZIF does not reach over 70%, indicating a possibly large amount of product formations other than CO and H_2_ (Fig. S13d). It implies that C–Ni–Co–ZIF samples could be more suitable for other CO_2_RR product generations than for syngas production.

## Conclusion

Atomically dispersed Fe-Co bimetallic catalysts are fabricated by introducing Fe into Co-ZIF along with the pyrolysis. The Fe-Co bimetallic catalysts show excellent activity toward syngas production from ECO_2_RR, with tunable CO/H_2_ ratios. C–Fe–Co–ZIF-1.6 wt%-Fe exhibits the highest FE_CO_ of 51.9% and FE_H2_ of 42.4% at − 0.55 V vs RHE, where the FE_CO_ is significantly increased (around 10% higher than that of pure C–Co–ZIF). The H_2_/CO ratio is tunable in a wide range from 0.8 to 4.2 while maintaining the total FE_CO+H2_ as high as 93% for more than 10 h. XAS technique greatly benefits the characterization of the bimetallic atomically dispersed Fe–Co catalysts. N K-edge and metal L-edge provide insights into N species and metal oxidation states in the catalysts. Metal K-edge offers detailed information in the coordination environments of the metal atoms. It confirms that the addition of Fe would interfere with the local coordination environment of Co in bimetallic catalysts and C–Fe–Co–ZIF-1.6 wt%-Fe has mild distortions in Co and Fe coordination environments. The proper amount of Fe in C–Fe–Co–ZIF-1.6 wt%-Fe increases the number of M–N sites and creates mild distortions in the local coordination environment of the metal sites, which is the key reason for the best CO production performance in ECO_2_RR among all the C–Fe–Co–ZIF samples. The excessive adding of Fe results in other product generation than CO and H_2_. Applied with the same strategy, atomically dispersed Cu–Co bimetallic catalysts also exhibit positive results on CO production for the syngas generation, while atomically dispersed Ni–Co bimetallic catalysts facilitate other product generations than syngas. The present results demonstrate the synergistic effects concerning the metal combination and the interactions between different metal atoms should be considered for the design of atomically dispersed TM bimetallic catalysts in ECO_2_RR.

## Supplementary Information

Below is the link to the electronic supplementary material.

Supplementary file1 (PDF 2120 KB)
