# Supplementary material for: Atomically Dispersed Fe-Co Bimetallic Catalysts for the Promoted Electroreduction of Carbon Dioxide
Source: Nanomicro Lett. 2021 Dec 10;14:25. doi: 10.1007/s40820-021-00746-9 (PMC8664923; doi:10.1007/s40820-021-00746-9)
Supplement: Supplementary file 1 — Supplementary file1 (PDF 2120 KB) [file 40820_2021_746_MOESM1_ESM.pdf]

Supporting information for

Atomically Dispersed Fe-Co Bimetallic Catalysts for the Promoted Electroreduction of Carbon Dioxide

Zhangsen Chen[1], Gaixia Zhang[1, *], Yuren Wen[2], Ning Chen[3], Weifeng Chen[3], Tom Regier[3], Jay Dynes[3], Yi Zheng[4, *], Shuhui Sun[1, *]

[1]Institut National de la Recherche Scientifique-Énergie Matériaux et Télécommunications, Varennes, Québec J3X 1S2, Canada

[2]School of Materials Science and Engineering, University of Science and Technology Beijing, 100083 Beijing, P. R. China

[3]Canadian Light Source, University of Saskatchewan, Saskatoon, SK S7N 2V3, Canada

[4]Research Institute of Photocatalysis, State Key Laboratory of Photocatalysis on Energy and Environment, Fuzhou University, Fuzhou 350116, P. R. China

*Corresponding authors. E-mail: gaixia.zhang@emt.inrs.ca (G. Zhang); yizheng@fzu.edu.cn (Y. Zheng); shuhui@emt.inrs.ca (S. Sun)

S1 Materials and Methods

S1.1 Chemicals

Cobalt nitrate hexahydrate ($Co(NO_3)_2 \cdot 6H_2O$, 98%, Sigma-Aldrich), zinc nitrate hexahydrate ($Zn(NO_3)_2 \cdot 6H_2O$, 98%, Fisher), 2-methylimidazole (2-mIm, 99%, Sigma-Aldrich), methanol (MeOH, 99.9%, Sigma-Aldrich), Iron (III) nitrate nonahydrate $Fe(NO_3)_3 \cdot 9H_2O$ (99.9%, Sigma-Aldrich), Nickel nitrate hexahydrate ($Ni(NO_3)_2 \cdot 6H_2O$, 98%, Sigma-Aldrich), Copper nitrate ($Cu(NO_3)_2 \cdot xH_2O$, 98%, Sigma-Aldrich), Potassium hydrogen carbonate ($KHCO_3$, 99%, Alfa Aesar) were used as received without any purification. Deionized water (18.2 MΩ·cm) was obtained from the ultrapure water system of Arium® Pro DI from SATORIUS.

S1.2 Synthesis of Co-ZIF

Typically, $Co(NO_3)_2 \cdot 6H_2O$ (0.546 g) and $Zn(NO_3)_2 \cdot 6H_2O$ (1.116 g) were dissolved in 40 mL of methanol. Then, 2-methylimidazole methanol solution (1.116 g in 40 mL) was slowly poured into the above solution under vigorously stirring, following with the subsequent stirring for 24 hours at room temperature. The as-obtained powders were collected by centrifugation at 7000 rpm for 5 minutes, washed three times with methanol, and finally dried in the oven at 90°C overnight.

S1.3 Synthesis of Fe-Co-ZIF with Different Fe Loadings

The powder of as-prepared Co-ZIF (100 mg) was dispersed in methanol (10 ml) under ultrasound for 5 min at room temperature. After forming homogeneous solution, $Fe(NO_3)_2 \cdot 9H_2O$ methanol solution (115 mg ml^{-1}, 50 µL for 0.8 wt% Fe, 100 µL for 1.6 wt% Fe, 200 µL for 3.2 wt% Fe, and 300 µL for 4.8 wt% Fe) was slowly injected into the mixed solution under ultrasound for 2 min at room temperature. Next, the mix solution was under vigorous stirring for 5 h at room temperature

to make the salt solution be absorbed completely. Then the samples were centrifuged and dried in the oven at 90°C overnight.

S1.4 Synthesis of C-Co-ZIF and C-Fe-Co-ZIFs

The powder of as-prepared Co-ZIF and Fe-Co-ZIFs were placed in the tube furnace, maintained at three temperature plateaus (800 °C, 900 °C, and 1000 °C, 1 hour for each) with a heating rate of 25 °C min^{-1} under flowing argon gas, and then naturally cooled to room temperature. All the as-prepared samples were directly used without any post-treatment.

S1.5 Synthesis of C-Cu-Co-ZIF and C-Ni-Co-ZIFs

The synthesis of C-Cu-Co-ZIF and C-Ni-Co-ZIFs follows the same procedure of C-Fe-Co-ZIF while replacing the $Fe(NO_3)_2$ methanol solution into the corresponding $Cu(NO_3)_2$ and $Ni(NO_3)_2$ methanol solution, respectively, during the ZIF preparation process.

S2 Supplementary Tables and Figures

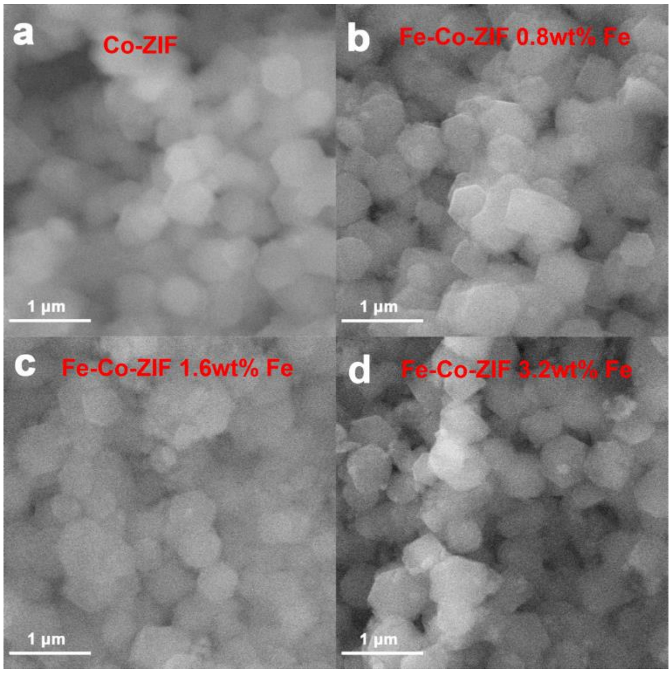

Fig. S1 SEM images of **a** Co-ZIF powders. **b** Fe-Co-ZIF-0.8 wt%-Fe powders. **c** Fe-Co-ZIF-1.6 wt%-Fe powders. **d** Fe-Co-ZIF-3.2 wt%-Fe powders

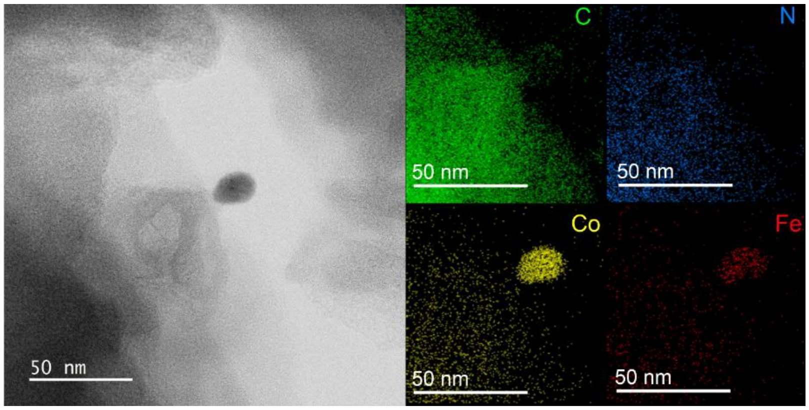

Fig. S2 TEM and EDX images of the clusters in the sample of C-Fe-Co-ZIF-3.2 wt%-Fe

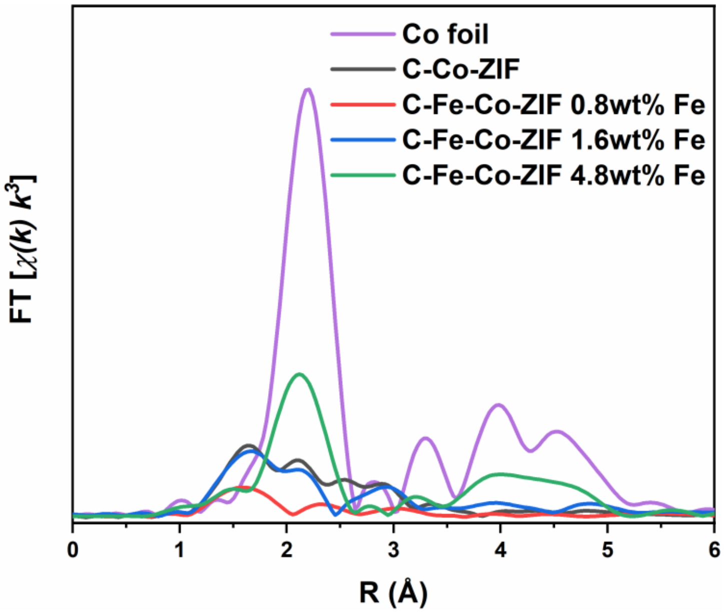

Fig. S3 EXAFS of Co foil, C-Co-ZIF, and C-Fe-Co-ZIF catalysts

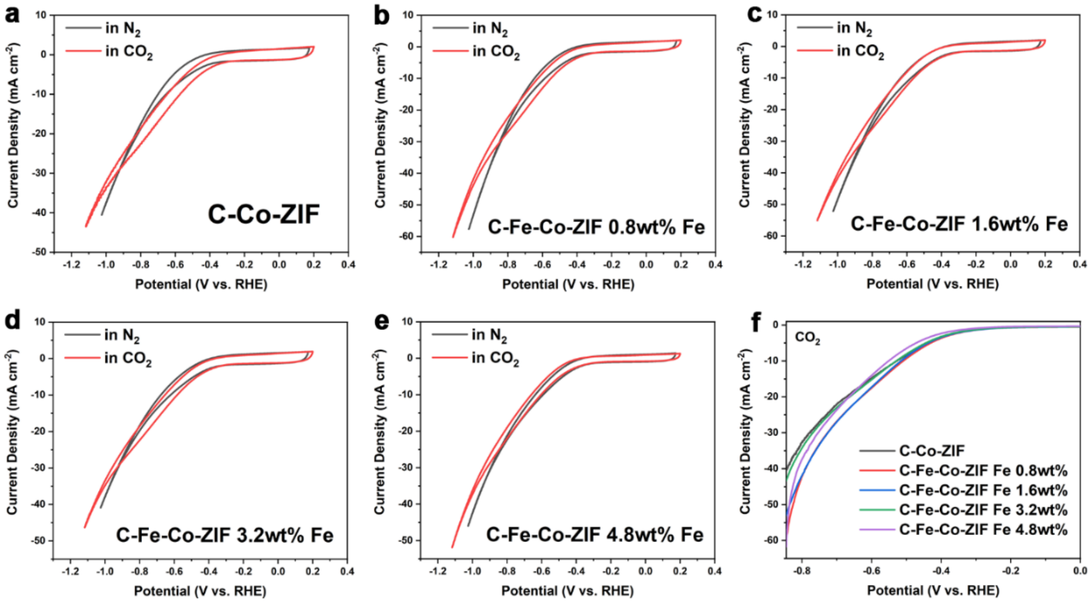

Fig. S4 Cyclic voltammograms of **a** C-Co-ZIF. **b** C-Fe-Co-ZIF-0.8 wt%-Fe. **c** C-Fe-Co-ZIF-1.6 wt%-Fe. **d** C-Fe-Co-ZIF-3.2 wt%-Fe. **e** C-Fe-Co-ZIF-4.8 wt%-Fe in CO_2-saturated 0.5 M $KHCO_3$ solution at a scan rate of 20 mV s^{-1} (no iR-compensation was made during CV tests). **f** LSV curves of the samples in CO_2-saturated 0.5 M $KHCO_3$ solution at a scan rate of 5 mV s^{-1} (80% iR-compensation)

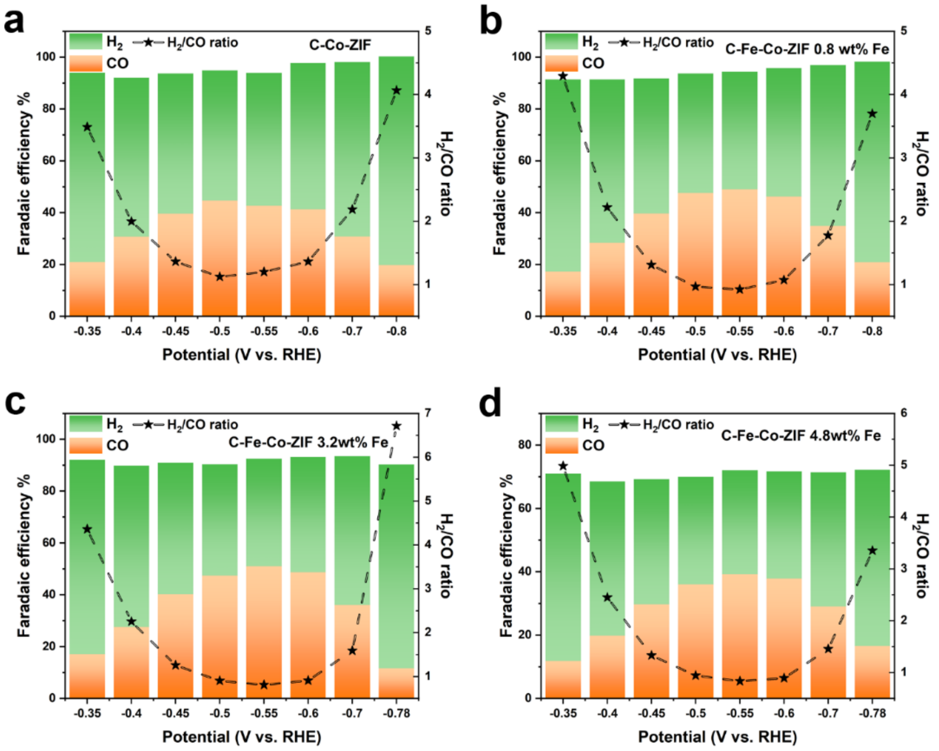

Fig. S5 Bars: FE_{CO} and FE_{H2}; Stars: H_2/CO ratio of **a** C-Co-ZIF. **b** C-Fe-Co-ZIF-0.8 wt%-Fe. **c** C-Fe-Co-ZIF-3.2 wt%-Fe. **d** C-Fe-Co-ZIF-4.8 wt%-Fe at various applied potentials

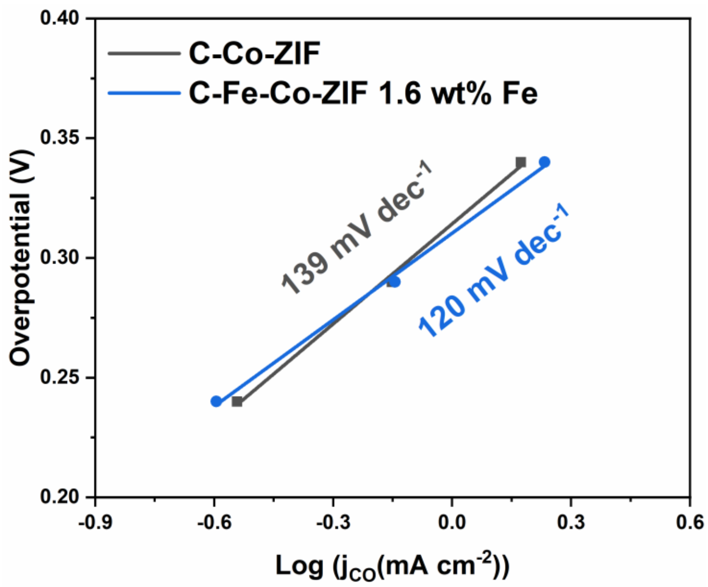

Fig. S6 Tafel slopes of C-Co-ZIF and C-Fe-Co-ZIF-1.6 wt%-Fe

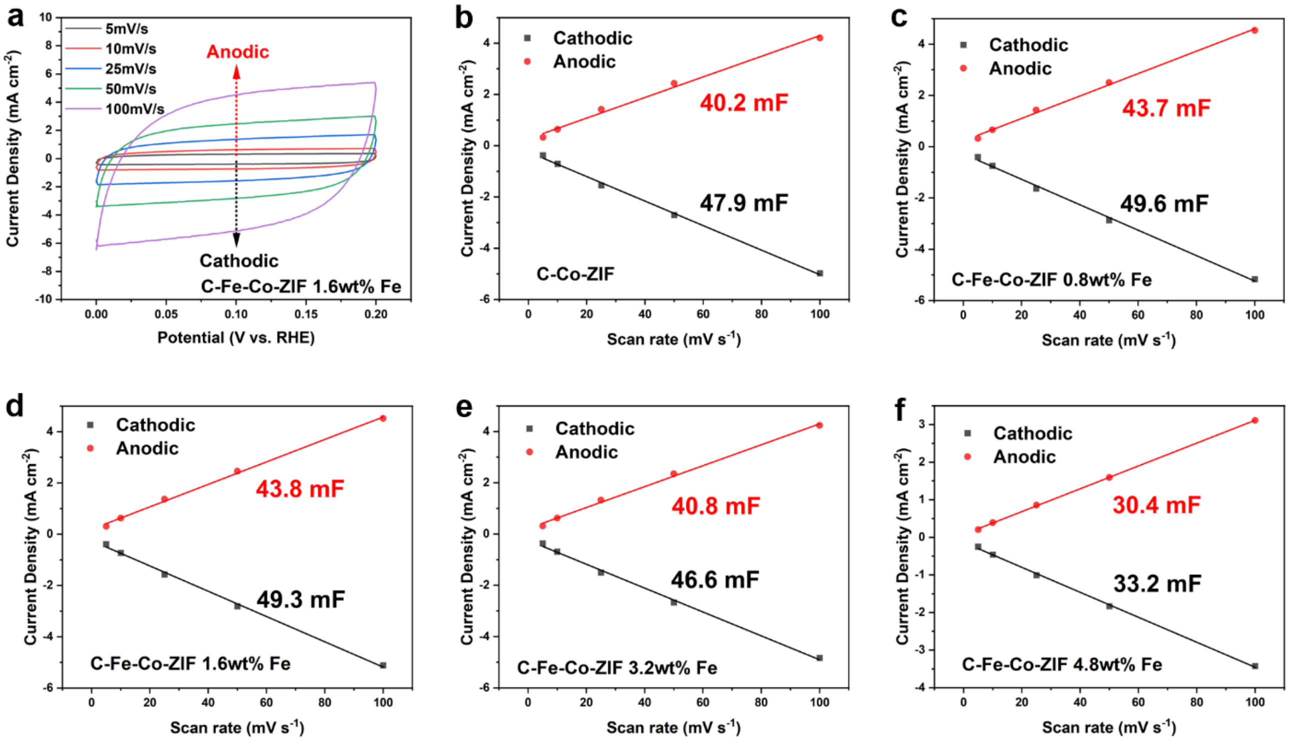

Fig. S7 Double-layer capacitance tests for evaluating the electrochemical active surface area of the catalysts **a** CVs of C-Fe-Co-ZIF-1.6 wt%Fe sample with scan rate from 5 mV s^{-1} to 100 mV s^{-1}. The cathodic (dark line) and anodic (red line) currents were measured at 0.10 V vs. RHE as a function of the scan rate of **b** C-Co-ZIF. **c** C-Fe-Co-ZIF-0.8 wt%-Fe. **d** C-Fe-Co-ZIF-1.6 wt%-Fe. **e** C-Fe-Co-ZIF-3.2 wt%-Fe. **f** C-Fe-Co-ZIF-4.8 wt%-Fe. The average value of the cathodic and anodic slopes is taken as the double-layer capacitance of the catalyst electrode.

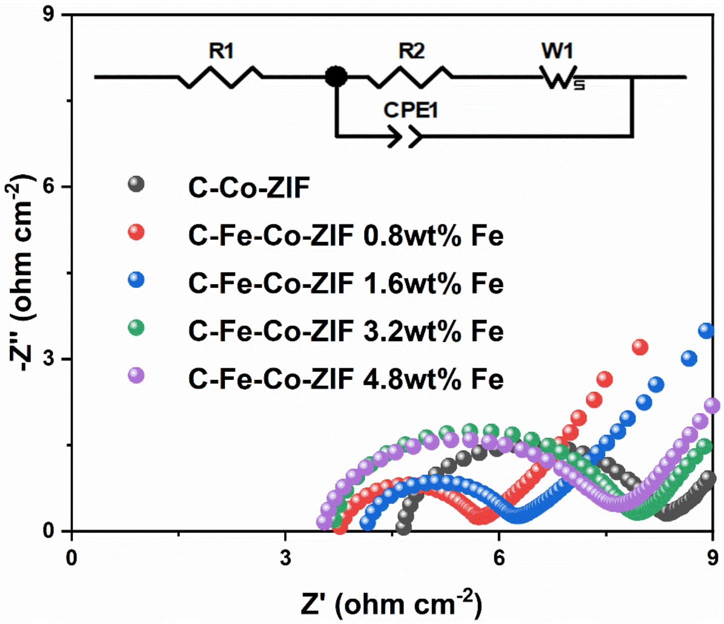

Fig. S8 EIS results of C-Co-ZIF and C-Fe-Co-ZIF catalysts

Table S1 Resistance of the catalysts at open circuit potential

	Resistance (Ω)
C-Co-ZIF	3.691
C-Fe-Co-ZIF-0.8 wt%-Fe	2.028
C-Fe-Co-ZIF-1.6 wt%-Fe	2.085
C-Fe-Co-ZIF-3.2 wt%-Fe	4.149
C-Fe-Co-ZIF-4.8 wt%-Fe	4.296

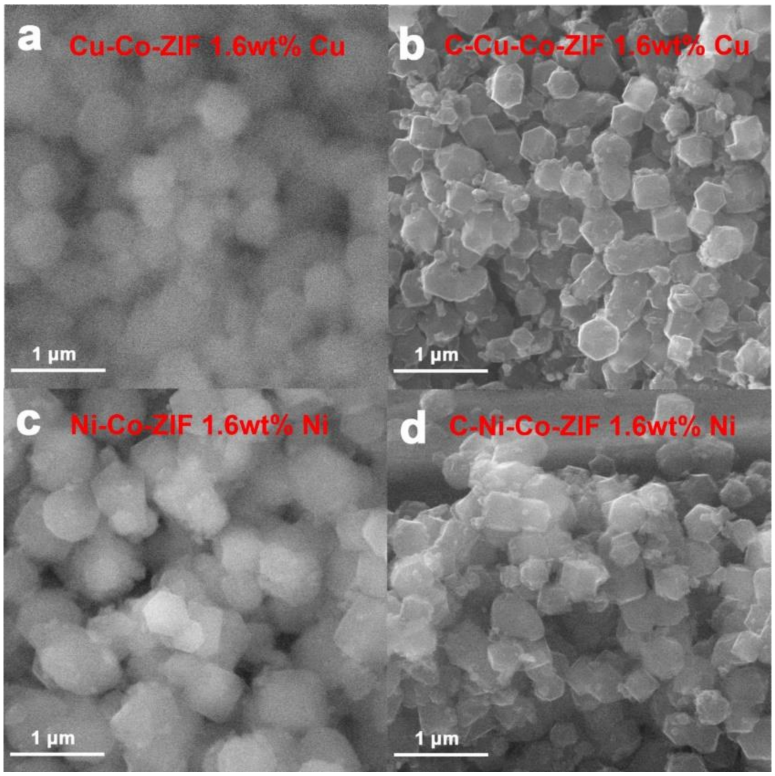

Fig. S9 SEM images of **a** Cu-Co-ZIF-1.6 wt%-Cu. **b** C-Cu-Co-ZIF-1.6 wt%-Cu. **c** Ni-Co-ZIF-1.6 wt%-Ni. **d** C-Ni-Co-ZIF-1.6 wt%-Ni

All the original and carbonized Cu-Co-ZIFs and Ni-Co-ZIFs have the same morphology with a particle size of around 300 nm. It illustrates that the series of Cu and Ni modified Co-ZIF samples retain the morphology of the original Co-ZIFs independent of the metal sources during the synthesis and pyrolysis processes.

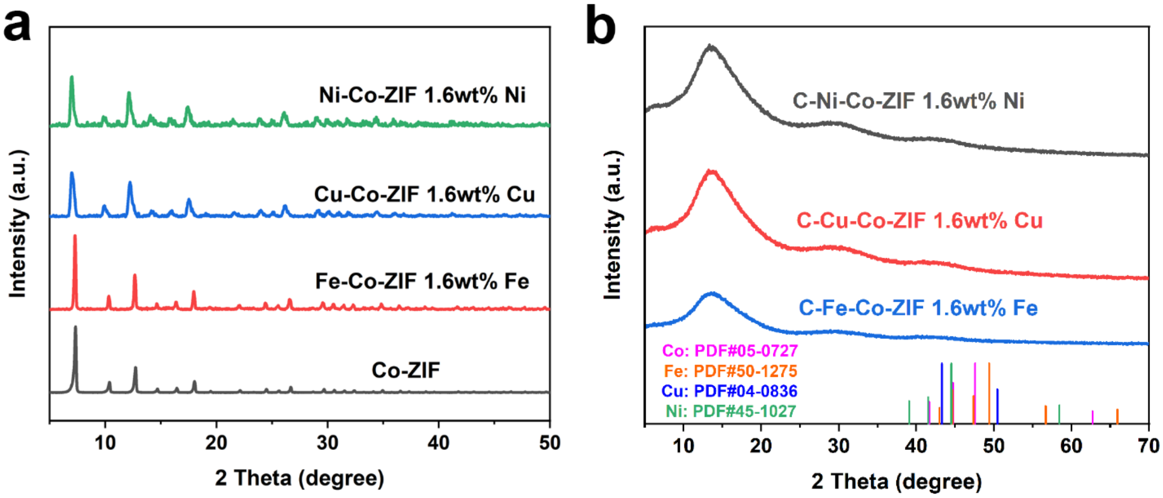

Fig. S10 XRD patterns of **a** M-Co-ZIF samples with M adding amount of 1.6 wt% (M=Ni, Cu, Co). **b** C-M-Co-ZIF samples with M adding amount of 1.6 wt% (M=Ni, Cu, Fe).

Cu-Co-ZIF and Ni-Co-ZIF share the same crystalline structure as that of Fe-Co-ZIF, without any obvious characteristic peak assigned to Cu and Ni crystals. It indicates that Cu-Co-ZIF and Ni-Co-ZIF keep the same crystalline structure as that of Co-ZIF as well. After the pyrolysis, carbonized Cu-Co-ZIF (C-Cu-Co-ZIF) and carbonized Ni-Co-ZIF (C-Cu-Co-ZIF) also appear to be the amorphous carbon material, same as C-Fe-Co-ZIF.

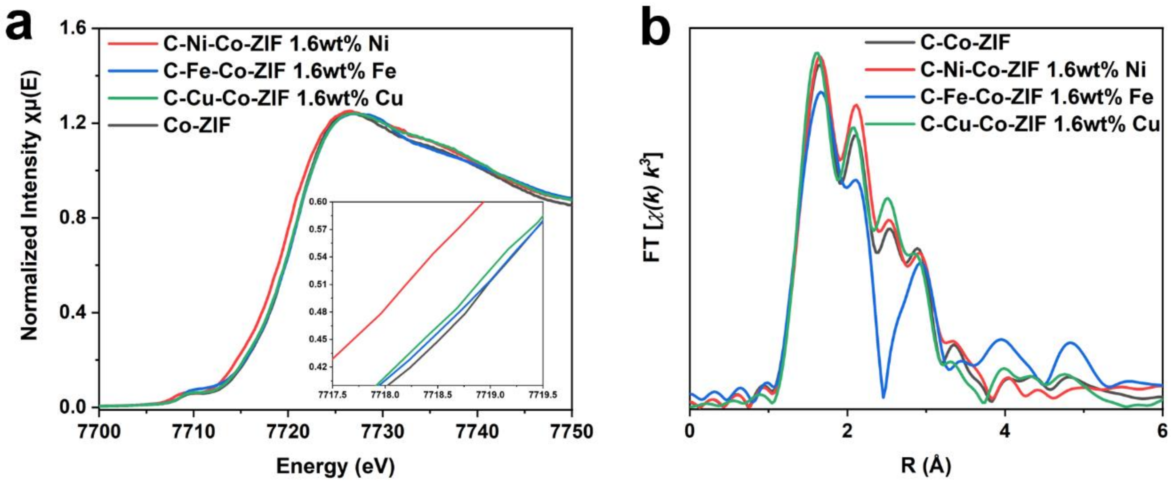

Fig. S11 a XANES (insert: enlarged white line region). **b** EXAFS of C-Co-ZIF and C-M-Co-ZIF samples with M adding amount of 1.6 wt% (M=Ni, Cu, Fe)

In **Fig. S11a**, the valances of Co atoms in C-Fe-Co-ZIF and C-Cu-Co-ZIF are close to that of C-Co-ZIF while the introduction of Ni reduces the valance of Co atoms in the catalyst, which is relatively greater than Fe and Cu. It implies The atomically dispersed state of the TM atoms and the interactions between Co and the introduced foreign TM atoms (Cu and Ni) are also confirmed by the analysis of EXAFS (**Fig. S11b**) where peaks at M-N and M-M regions appear for both C-Cu-Co-ZIF and C-Ni-Co-ZIF. Compared to C-Fe-Co-ZIF, the Co *R* space patterns in C-Cu-Co-ZIF and C-Ni-Co-ZIF are closer to that of C-Co-ZIF.

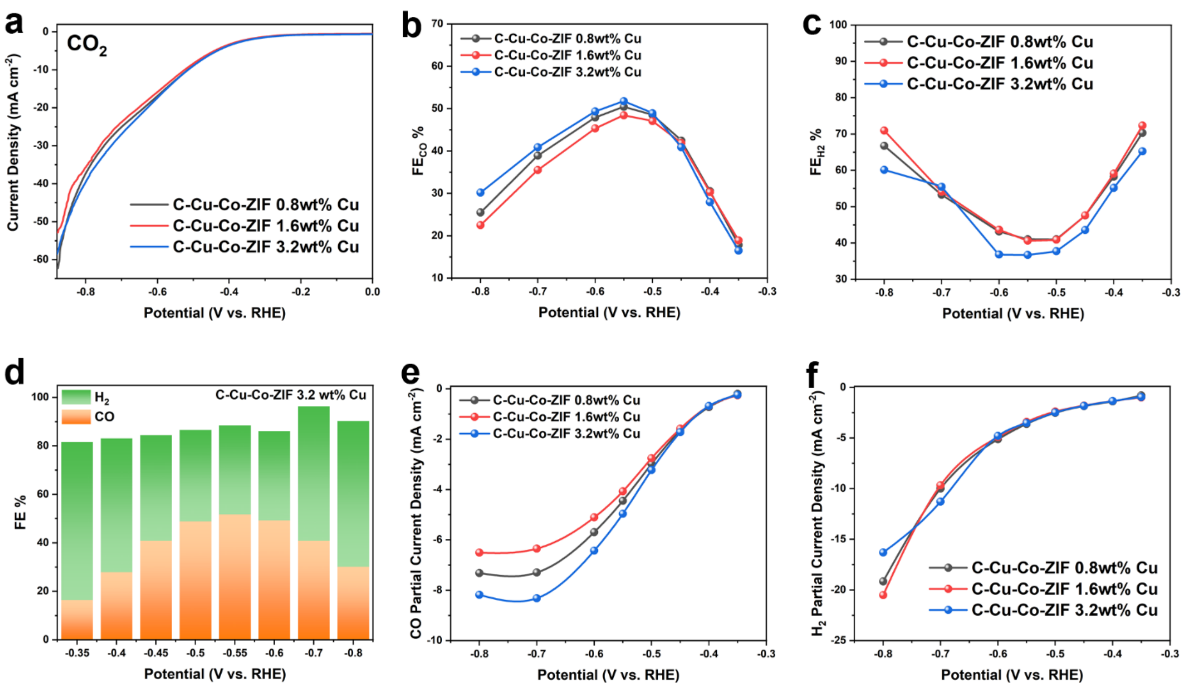

Fig. S12 The evaluation of the electrocatalytic performance of C-Cu-Co-ZIF with different Cu adding amounts. **a** LSV curves in CO_2-saturated 0.5 M $KHCO_3$ solution at a scan rate of 5 mV s^{-1}. **b** CO Faradaic efficiency of the catalysts at various applied potentials. **c** H_2 Faradaic efficiency of the catalysts at various applied potentials. **d** Total Faradaic efficiency of CO and H_2 of C-Cu-Co-ZIF-3.2 wt%-Cu at various applied potentials. **e** CO current density of the catalysts. **f** H_2 current density of the catalysts.

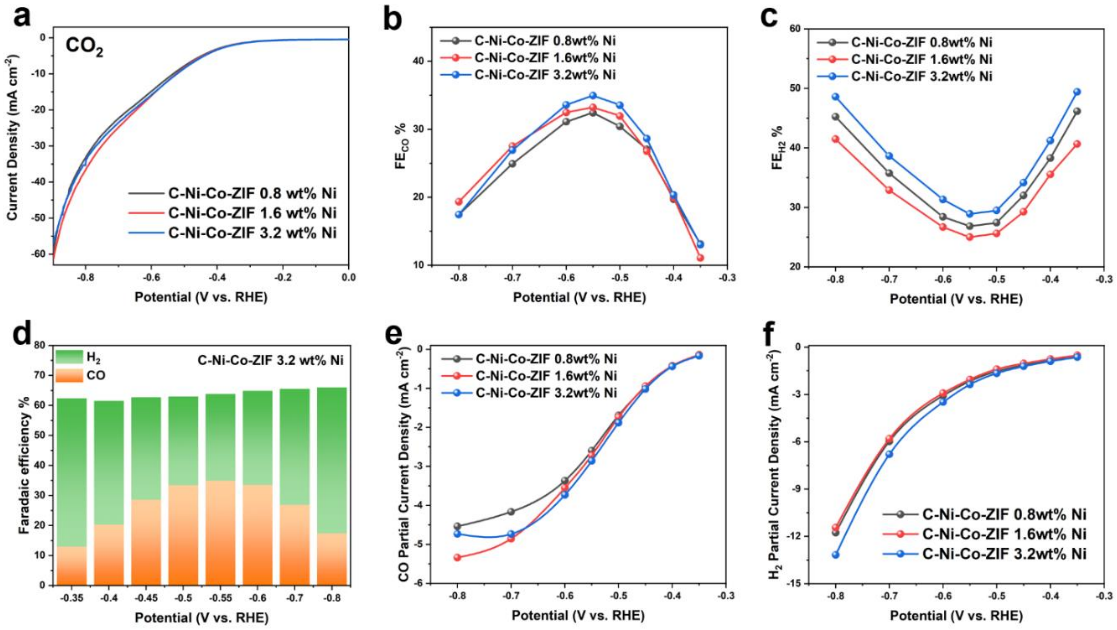

Fig. S13 The evaluation of the electrocatalytic performance of C-Ni-Co-ZIF with different Ni adding amounts. **a** LSV curves in CO_2-saturated 0.5 M $KHCO_3$ solution at a scan rate of 5 mV s^{-1}. **b** CO Faradaic efficiency of the catalysts at various applied potentials. **c**) H_2 Faradaic efficiency

of the catalysts at various applied potentials. **d** Total Faradaic efficiency of CO and H_2 of C-Ni-Co-ZIF-3.2wt%-Ni at various applied potentials. **e** CO current density of the catalysts. **f)** H_2 current density of the catalysts.

Table S2 CO_2RR performances of diverse electrocatalysts

Catalyst	FE	Current density (mA cm^{-2})	Potential (overpotential) vs. RHE	Medium	Refs.
topological defect-enriched porous carbons (PC-TD)	FE_{CO} 99.0%	0.37	-0.4 V (283 mV)	0.5 M $KHCO_3$	[S1]
A-Ni-NSG	FE_{CO} 94.0%	23.5	-0.72 V (610 mV)	0.5 M $KHCO_3$	[S2]
high-spin-state nickel(II) phthalocyanine (HS-NiPc)	FE_{CO} 98.5%	2.7	-0.7 V (590 mV)	0.5 M $KHCO_3$	[S3]
Ni-N_3S	FE_{CO} 95.0%	7.8	-0.8 V (690 mV)	0.5 M $KHCO_3$	[S4]
Ni@NCNTs-CNF	FE_{CO+H2} 100.0%	25	-0.8 V	0.5 M $KHCO_3$	[S5]
CoPc	FE_{CO} 99.0%	8.0	-0.8 V (690 mV)	0.5 M $KHCO_3$	[S6]
Co-N_2	FE_{CO} 94%	18.1	-0.63 V (520 mV)	0.5 M $KHCO_3$	[S7]
Fe/NG	FE_{CO} 80%	2.0	-0.57 V (460 mV)	0.1 M $KHCO_3$	[S8]
Fe-N-C	FE_{CO} >80%	25.0	-0.47 V (360 mV)	0.5 M $KHCO_3$	[S9]
Pd/NbN	FE_{CO+H2} ~90.0%	~1.0	-0.6 V	0.5 M $NaHCO_3$	[S10]
CoNi-NC	FE_{CO+H2} ~99.0%	50	-0.9 V	0.5 M $KHCO_3$	[S11]
C-Fe-Co-ZIF	FE_{CO} 51.9% FE_{H2} 42.4%	8.7	-0.55 V	0.5 M $KHCO_3$	This work

Supplementary References

[S1] P. Jiang, K. Jiang, D. Tranca, J. Zhu, F. Qiu et al., Rational control of topological defects in porous carbon for high-efficiency carbon dioxide conversion. Adv. Mater. Interfaces **8**(7), 2100051 (2021). https://doi.org/10.1002/admi.202100051

[S2] H.B. Yang, S.-F. Hung, S. Liu, K. Yuan, S. Miao et al., Atomically dispersed Ni(I) as the active site for electrochemical CO_2 reduction. Nat. Energy **3**(2), 140-147 (2018). https://doi.org/10.1038/s41560-017-0078-8

[S3] X. Wang, Y. Fu, D. Tranca, K. Jiang, J. Zhu et al., Regulating the spin state of nickel in molecular catalysts for boosting carbon dioxide reduction. ACS Appl. Energy Mater. **4**(3), 2891-2898 (2021). https://doi.org/10.1021/acsaem.1c00269

[S4] X. Zhao, S. Huang, Z. Chen, C. Lu, S. Han et al., Carbon nanosheets supporting Ni–N_3S single-atom sites for efficient electrocatalytic CO_2 reduction. Carbon **178**(488-496 (2021). https://doi.org/10.1016/j.carbon.2021.03.017

[S5] S.J. Shen, C. Han, B. Wang, Y.A. Du, Y.D. Wang, Dual active sites-dependent syngas proportions from aqueous CO_2 electroreduction. Appl. Catal. B-Environ. **279**(119380 (2020). https://doi.org/10.1016/j.apcatb.2020.119380

[S6] Z. Zhang, J. Xiao, X.J. Chen, S. Yu, L. Yu et al., Reaction mechanisms of well-defined metal-N_4 sites in electrocatalytic CO_2 reduction. Angew. Chem. Int. Ed. **57**(50), 16339-16342 (2018). https://doi.org/10.1002/anie.201808593

[S7] X. Wang, Z. Chen, X. Zhao, T. Yao, W. Chen et al., Regulation of coordination number over single Co sites: Triggering the efficient electroreduction of CO_2. Angew. Chem. Int. Ed. **57**(7), 1944-1948 (2018). https://doi.org/10.1002/anie.201712451

[S8] C. Zhang, S. Yang, J. Wu, M. Liu, S. Yazdi et al., Electrochemical CO_2 reduction with atomic iron-dispersed on nitrogen-doped graphene. Adv. Energy Mater. **8**(19), 1703487 (2018). https://doi.org/10.1002/aenm.201703487

[S9] J. Gu, C.S. Hsu, L. Bai, H.M. Chen, X. Hu. Atomically dispersed Fe^{3+} sites catalyze efficient CO_2 electroreduction to CO. Science **364**(6445), 1091-1094 (2019). https://doi.org/10.1126/science.aaw7515

[S10] Y. Liu, D. Tian, A.N. Biswas, Z. Xie, S. Hwang et al., Transition metal nitrides as promising catalyst supports for tuning CO/H_2 syngas production from electrochemical CO_2 reduction. Angew. Chem. Int. Ed. **59**(28), 11345-11348 (2020). https://doi.org/10.1002/anie.202003625

[S11] Q. He, D. Liu, J.H. Lee, Y. Liu, Z. Xie et al., Electrochemical conversion of CO_2 to syngas with controllable CO/H_2 ratios over Co and Ni single-atom catalysts. Angew. Chem. Int. Ed. **59**, 3033-3037 (2019). https://doi.org/10.1002/anie.201912719
